# Toward a storage ring coherent light source based on an angular dispersion-induced microbunching scheme

**DOI:** 10.1107/S1600577524010907

**Published:** 2025-01-01

**Authors:** Changliang Li, Bocheng Jiang, Chao Feng, Qiang Gu, Zhenghe Bai, Weishi Wan, Qinglei Zhang, Kun Wang

**Affiliations:** a Shanghai Advanced Research Institute Chinese Academy of Sciences Shanghai201204 People’s Republic of China; b Laboratory for Ultrafast Transient Facility Chongqing University Chongqing401331 People’s Republic of China; c National Synchrotron Radiation Laboratory USTC Hefei230029 People’s Republic of China; d School of Physical Science and Technology ShanghaiTech University Shanghai201210 People’s Republic of China; e Shanghai Institute of Applied Physics Chinese Academy of Sciences Shanghai201800 People’s Republic of China; f University of Chinese Academy of Sciences Beijing100049 People’s Republic of China; RIKEN SPring-8 Center, Japan

**Keywords:** storage rings, angular dispersion modulation, dispersion-induced microbunching, modulation–demodulation section, coherent radiation, damping wigglers

## Abstract

The combination of reversible angular dispersion-induced microbunching (ADM) and the rapid damping storage ring provides a storage-ring-based light source with the capability to produce longitudinal coherent radiation with a high repetition rate. This paper presents a prototype design for a test facility based on the study by Jiang *et al.* [*Sci. Rep.* (2022), **12**, 3325]. The modulation–demodulation section is inserted into a long straight section of the storage ring instead of a bypass line, which poses great challenges for the optimization of the nonlinear dynamics of the storage ring. However, this design avoids the challenging injection and extraction system connecting to the bypass line. To utilize mature laser technology and reduce the difficulty of the reversible ADM lattice design, we use a long-wavelength 1030 nm seed laser. In the simulation, we achieved 20th harmonic radiation with a bunching factor of about 7.2%. The growth rate of vertical emittance and energy spread of the electron beam for a single pass are about 11% and 0.02%, respectively. When the energy of the electron beam is 800 MeV and two sets of damping wigglers are employed, the damping time in the vertical plane is reduced to 8.31 ms. This results in a 438 kHz repetition rate of the coherent radiation at the new equilibrium state.

## Abstract 2

In this work, a storage-ring-based light source is developed to generate longitudinal coherent radiation at a high repetition rate as a result of the combination of a rapid damping storage ring and reversible angular dispersion-induced microbunching.

## 1. Introduction

Extreme ultraviolet (EUV) lithography is a topic of great interest to the accelerator community. Various schemes have been proposed for EUV lithography, including steady-state microbunching (SSMB) (Deng *et al.*, 2021 ▸; Zhang *et al.*, 2021 ▸), storage-ring-based FEL (Lee *et al.*, 2020 ▸; Di Mitri *et al.*, 2021 ▸) and ERL-based FEL (Zhao *et al.*, 2021 ▸; Nakamura *et al.*, 2023 ▸; Socol *et al.*, 2011 ▸; Venturini & Penn, 2015 ▸) *etc*. Proof-of-principle experiments have been carried out for SSMB, and one of the most important features for this scenario is changing the longitudinal focusing system from the radiofrequency (RF) cavity to the laser modulator. This creates electron beam bunches with lengths less than 100 nm throughout the storage ring. To achieve the extremely short bunch, not only alpha but also partial alpha should be maintained at very small values. To generate EUV radiation, it is essential to minimize the bunch length to a few nanometres, which requires the implementation of longitudinal strong focusing or angular dispersion-induced microbunching (ADM) (Feng & Zhao, 2017 ▸; Li *et al.*, 2020 ▸; Lu *et al.*, 2022 ▸; Li *et al.*, 2021 ▸; Li *et al.*, 2022 ▸) to effectively suppress the bunch length prior to the radiator. The ring-based FEL includes seeded FEL which mainly refers to high-gain harmonic generation (HGHG) (Yu, 1991 ▸; Yu *et al.*, 2000 ▸) and echo-enabled harmonic generation (EEHG) (Stupakov, 2009 ▸; Xiang & Stupakov, 2009 ▸; Büsing *et al.*, 2022 ▸). The HGHG scheme typically generates harmonic numbers that do not exceed 10 primarily due to the large energy spread of the beam in the storage ring. EEHG simulation in the storage ring has been conducted showing the harmonic number can reach higher values (Yang *et al.*, 2022 ▸; Yang *et al.*, 2023*a* ▸; Yang *et al.*, 2023*b* ▸). However, a high-power laser is required for the EEHG experiment carried out at the DELTA storage ring, and limited harmonic numbers were achieved (Büsing *et al.*, 2022 ▸). There is a ring-based self-amplified spontaneous emission (Kondratenko & Saldin, 1980 ▸) FEL scheme that does not require a seed laser by employing transverse gradient undulators (TGUs) (Di Mitri *et al.*, 2021 ▸). The use of TGUs can reduce the broadening of the resonance wavelength range due to energy spread and avoid a sharp drop in FEL gain. This scheme generally requires an undulator with a length of nearly 100 m, and its repetition rate is relatively low, about few kilohertz.

The ADM scheme generates high harmonics with a seed laser power reduced by approximately an order of magnitude compared with the other schemes mentioned above (Feng & Zhao, 2017 ▸). Additionally, the lattice structure of ADM is simple and does not require two energy modulations as in the case of the EEHG. The ADM scheme can also be applied to the ERL for generating high-power EUV radiation and the related design was given by Zhao *et al.* (2021 ▸), where simulations show that EUV radiation can reach up to about 100 W.

The discovery of the transparent damping wiggler (DW) makes it possible to apply the ADM scheme to low-energy electron storage rings to realize high-power EUV radiation by increasing the repetition rate (Jiang *et al.*, 2022 ▸). The transparent DW inserted in the storage ring can cancel each other’s nonlinearities between periods due to the π nodes contained inside, which can greatly reduce the nonlinear dynamics of the storage ring. The combination of transparent DW and the demodulation scheme can significantly increase the repetition rate of the coherent radiation. However, the modulation–demodulation section (MDS) presents a great challenge for optimizing the nonlinear dynamics in the storage ring owing to its complex constraints. In Jiang *et al.* (2022 ▸), we make the MDS into a bypass line so that the ring will not affect the nonlinear dynamics from the MDS. However, injecting and extracting the electron beam into and out of the bypass line poses high demands on the hardware systems.

This paper presents a lattice design of a storage ring for a test facility that aims to reduce stress on the hardware by directly inserting the MDS into the long straight section (LSS). The lattice design includes the bare ring, the LSS and the MDS. Nonlinear dynamics of the full ring are optimized and simulations are conducted to demonstrate the performance of the MDS. We provide the design of a full storage ring lattice in Section 2. In Section 3, we present the simulation results of the reversible ADM. In Section 4, we improve the coherent radiation repetition rate by adding DW and increasing the beam energy. Conclusions are given in Section 5.

## 2. Storage ring lattice design

When the electron beam passes through the transport line, its longitudinal position changes as
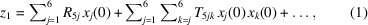
where *x*_1_ = *x*, *x*_2_ = *x*′, *x*_3_ = *y*, *x*_4_ = *y*′, *x*_5_ = *z*, *x*_6_ = δ and *x*_*i*_ represent the six-dimensional coordinates of the electron beam. Note that only the second-order transport effects are considered here; higher-order effects are neglected for simplicity. According to equation (1), when the emittance of the electron beam is larger, the greater the impact of the high-order term *T*_5*jk*_ on the longitudinal position shift will be. In previous studies (Jiang *et al.*, 2022 ▸), it was demonstrated that a longitudinal position shift between the modulator and the demodulator leads to an increase in energy spread and vertical emittance. To mitigate these effects, it is essential not only to set *R*_56_ to zero but also to minimize high-order terms. A low-emittance storage ring significantly facilitates the MDS design, as the impact of terms such as *R*_51_, *R*_52_, *T*_511_, *T*_512_*etc.* can be effectively suppressed.

The ADM scheme requires energy modulation of the electron beam in the storage ring. Since the vertical dispersion at the modulator is non-zero, the energy modulation is mapped to the vertical plane, which leads to an increase in the vertical emittance. An imperfect energy demodulation will leave an increased energy spread as well as vertical emittance. Due to the larger increases of the vertical emittance and the energy spread compared with the reductions caused by radiation damping, turn-by-turn-based operation will be constrained. There is a high demand of a shorter damping time to enhance the repetition rate.

**Figure 1:**
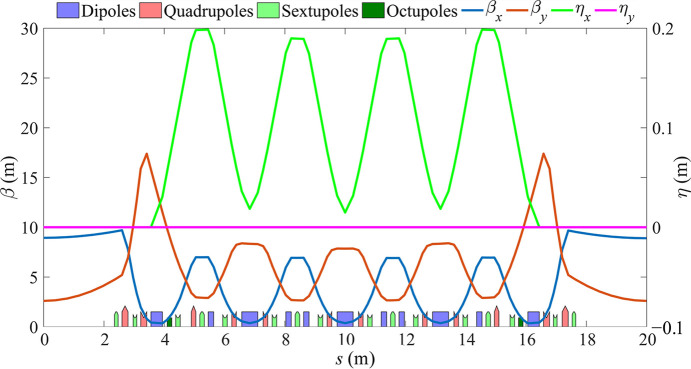
Optical functions for one 5BA standard cell.

The natural emittance of the electron beam can be reduced by adopting a multi-bend achromat lattice (Einfeld *et al.*, 2014 ▸; Tavares *et al.*, 2014 ▸; Tavares *et al.*, 2018 ▸). In this work, we use the lattice structure of a 5BA higher-order achromat (HOA) and employ reverse bends to reduce the emittance and enhance the radiation damping (Streun, 2014 ▸; Streun *et al.*, 2023 ▸). A 5BA lattice has been designed with an energy of 600 MeV (with the ability to increase the magnetic field strength to 800 MeV), a circumference of 160 m and six standard cells, with a natural emittance of 498 pm rad. The unit cell tunes of the 5BA lattice are about (0.4, 0.1) to make an HOA for effective sextupole cancelation (Bengtsson & Streun, 2017 ▸; Yang *et al.*, 2021 ▸). Fig. 1 ▸ displays the linear optics of a standard cell of the storage ring. The magnets contained in a standard cell are 5 normal bends, 6 reverse bends, 12 quadrupoles, 16 sextupoles and 2 octupoles.

**Figure 2:**
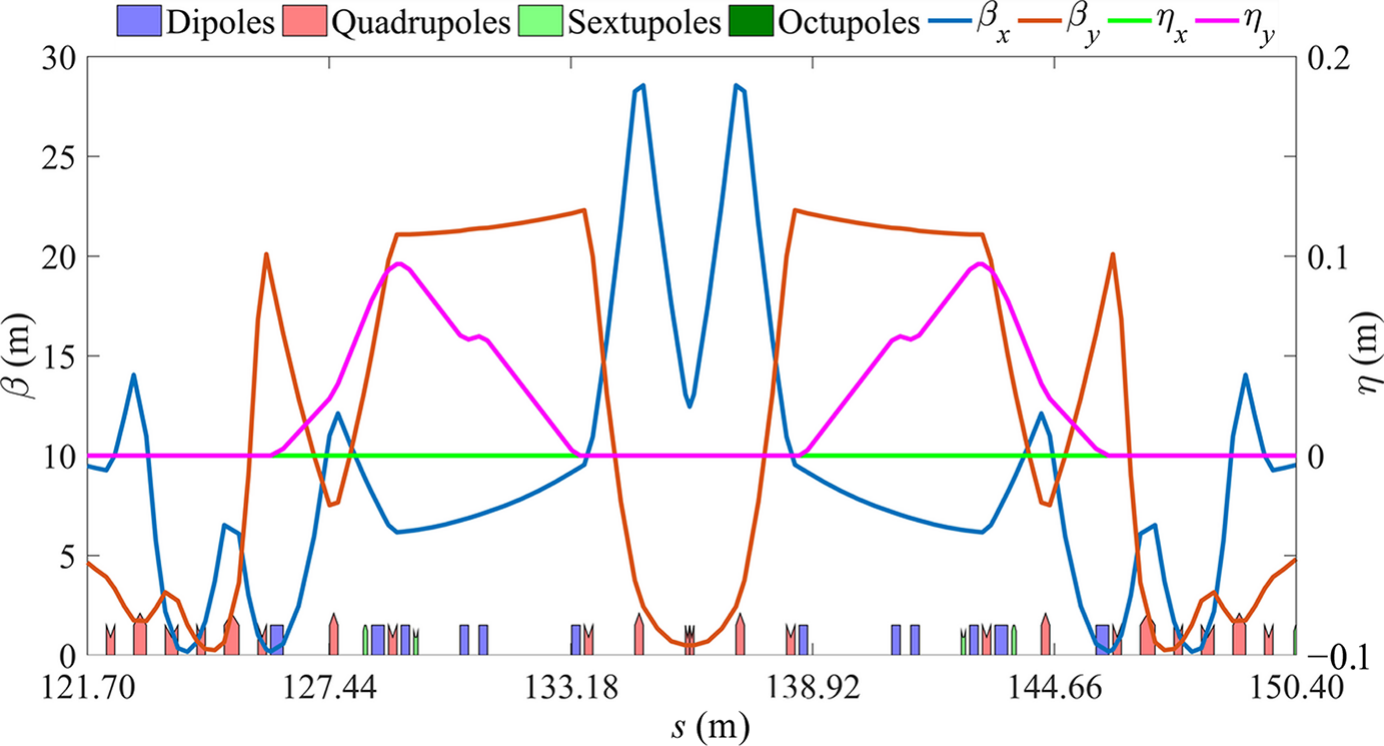
Optical functions for the MDS.

The design principle of the MDS was presented by Jiang *et al.* (2022 ▸). This paper differs from Jiang *et al.* (2022 ▸) in that the MDS is inserted into the LSS of the storage ring instead of the bypass. The vertical beta function at the entrance of the first vertical dipole is reduced in this case to reduce the natural chromaticity created by the MDS which will benefit nonlinear optimization. The bunching factor is then decreased. The Twiss parameters of the MDS are matched to the first harmonic sextupoles at the end of the straight section. Fig. 2 ▸ shows the optical functions of the MDS, with the maximum beta function controlled within 30 m.

**Figure 3:**
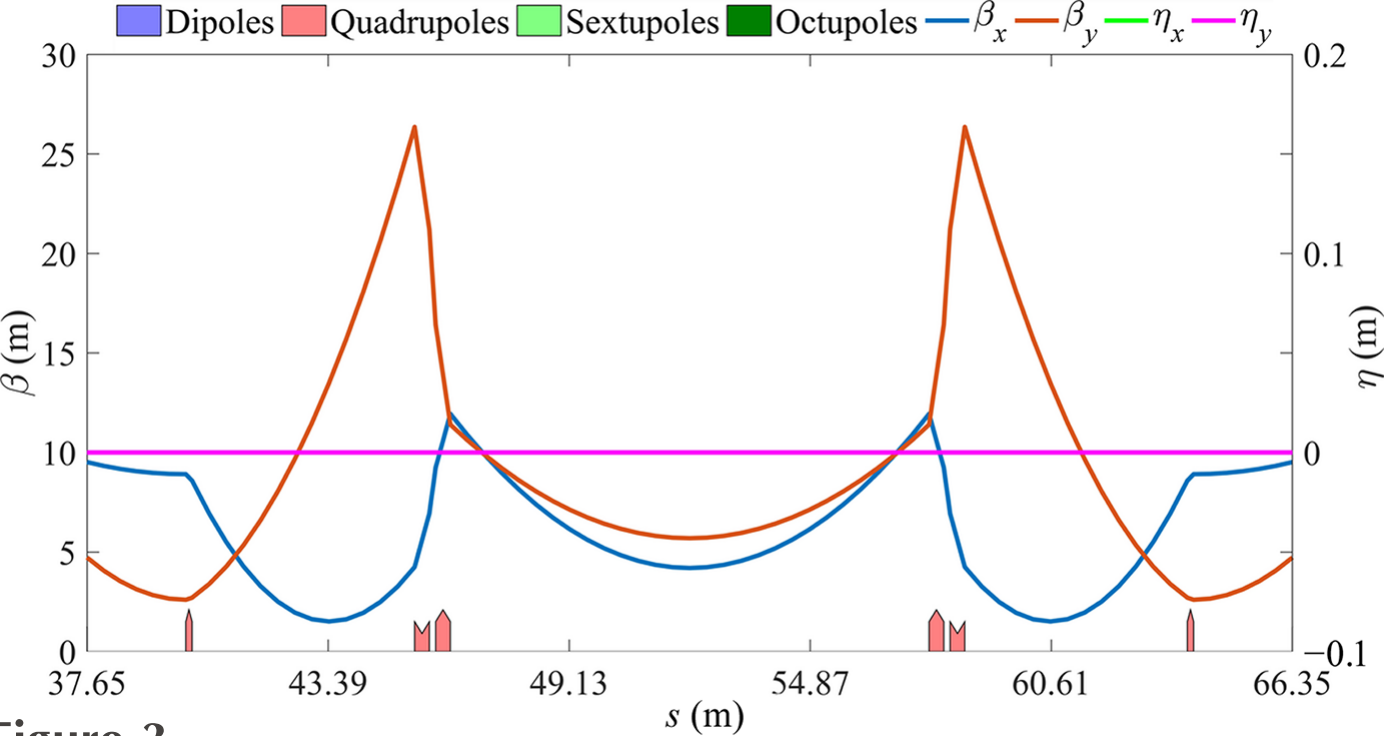
Optical functions for the LSS.

The total length of the MDS is 28.7 m. The same length of LSS must be placed on the opposite side of the ring. This LSS lattice is composed of three families of quadrupole magnets (six total) symmetrically arranged, leaving enough space in the straight section for the placement of other elements, such as the DW, which will be discussed in the next section. Fig. 3 ▸ shows the optical functions of the LSS, with the longest drift in the center measuring 11.4 m.

**Figure 4:**
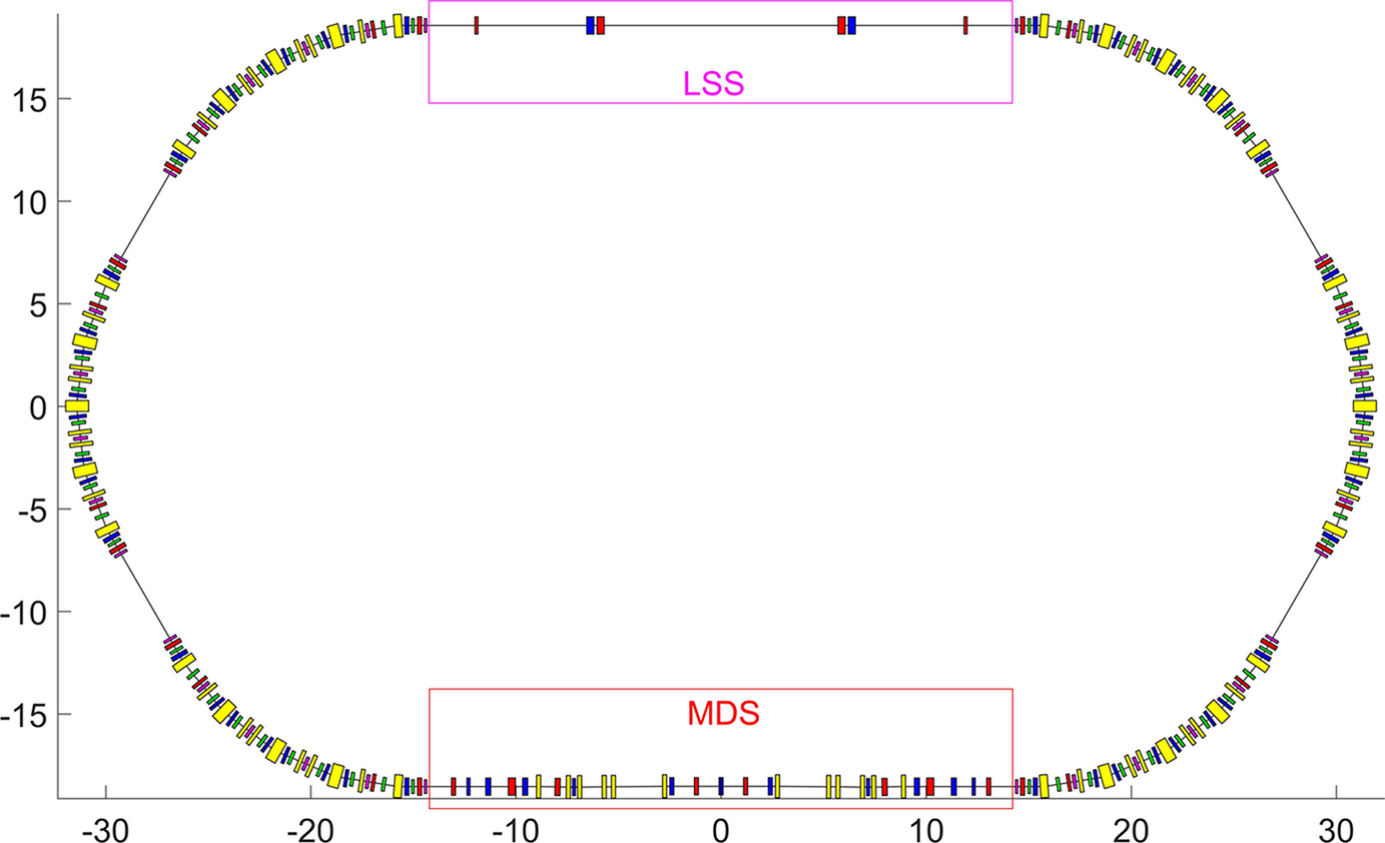
Layout of the full ring.

**Figure 5:**
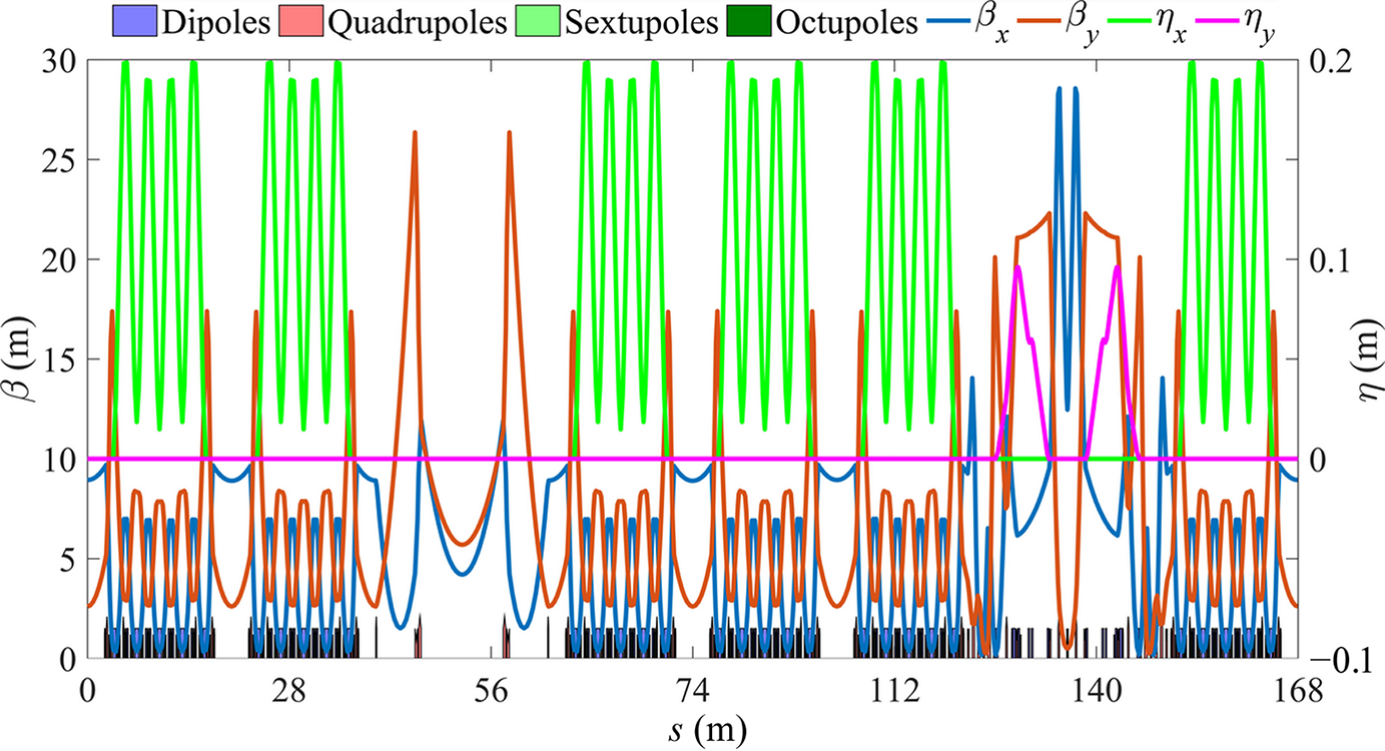
Optical functions for the full ring.

The storage ring comprises four standard straight sections, each with a length of 4.7 m, in addition to the 28.7 m-long LSS and MDS. The total circumference of the ring is 168 m. Fig. 4 ▸ shows the overall layout and Fig. 5 ▸ shows the linear optics of the full ring; Table 1 ▸ presents the beam parameters of the storage ring.

**Figure 6:**
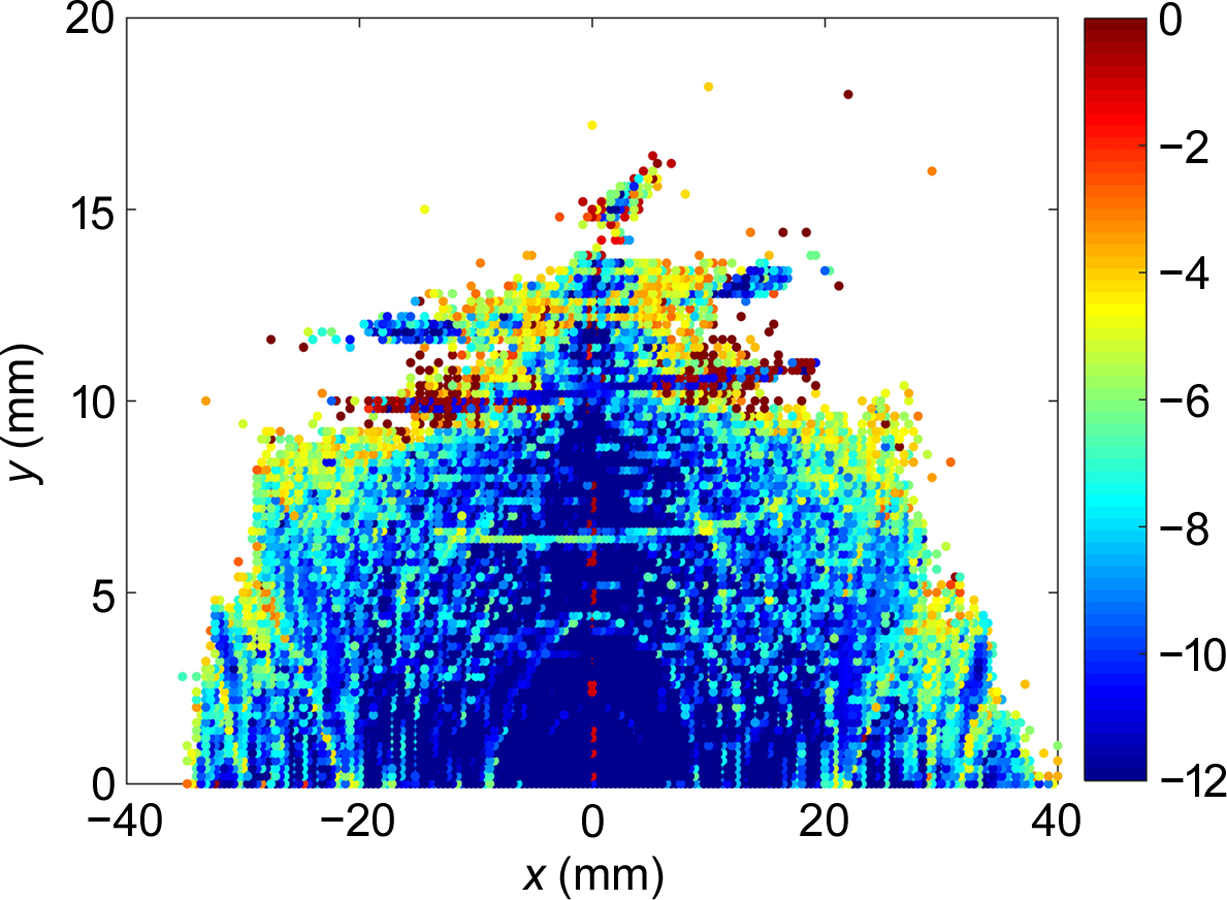
DA. The color bar represents the diffusion rate *D*_r_ for regular (blue) and chaotic (red) motion.

**Figure 7:**
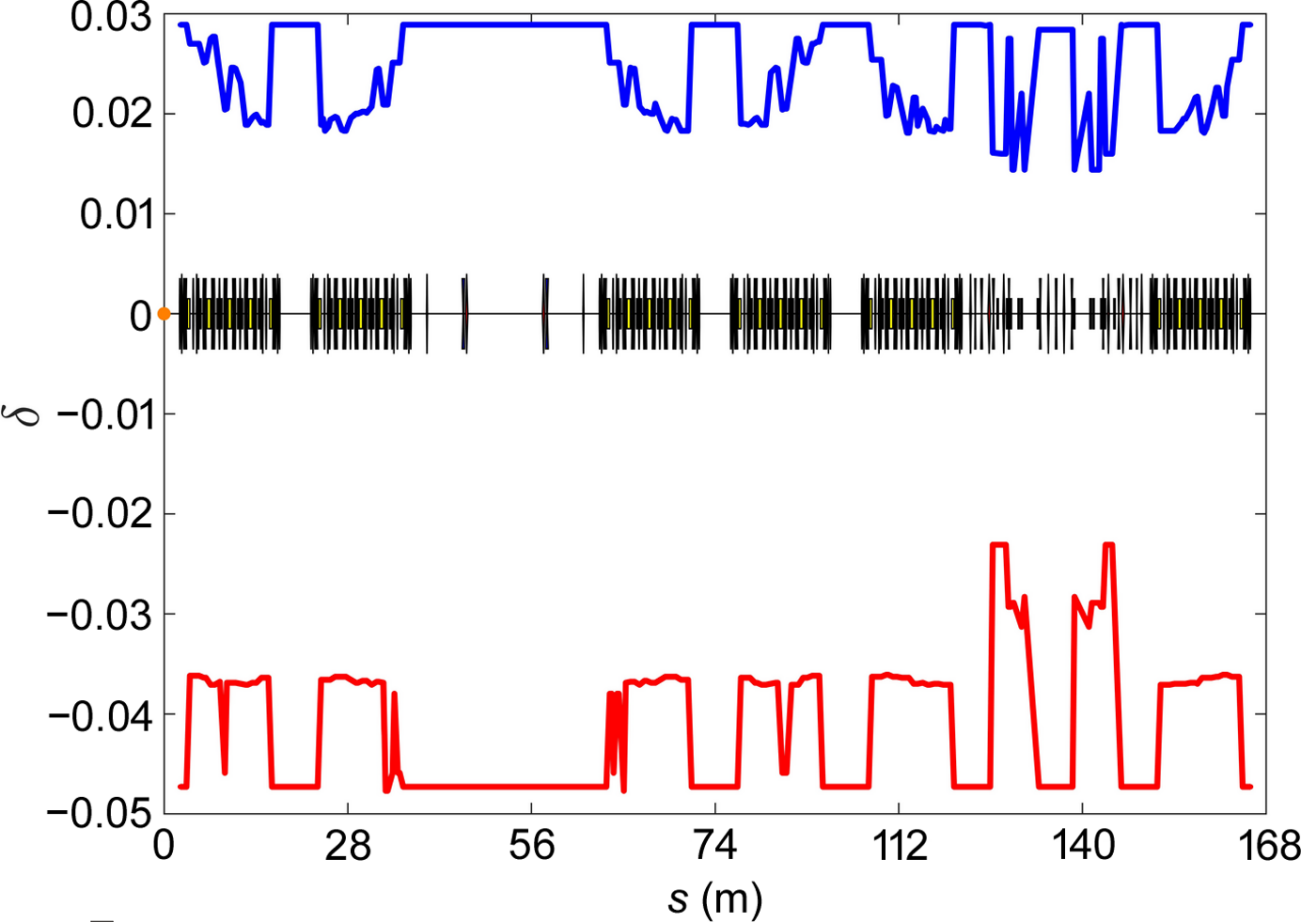
Local momentum acceptance.

The sixfold periodicity of the ring is broken by the LSS and MDS, and the transverse nonlinear beam dynamics optimization is a challenging task. The full ring contains four families of sextupoles: two families of chromatic sextupoles and two families of harmonic sextupoles. Through multiple iterative optimizations between reducing resonance driving terms and tracking verification (Yang *et al.*, 2011 ▸; Wei *et al.*, 2023 ▸), the dynamic aperture (DA) at the injection point is obtained as shown in Fig. 6 ▸, and the local momentum acceptance (LMA) of the full ring is shown in Fig. 7 ▸. Fig. 6 ▸ shows that the DA is large enough to allow for off-axis injection of the beam. The diffusion rate is defined as 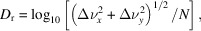
where Δν_*x*_ and Δν_*y*_ are the differences in *x* and *y* tunes from the first and second half of the tracking, respectively, and *N* is the number of turns. A smaller *D*_r_ indicates more stable particle motion. The fluctuations of the LMA agree with the predictions, with a minimum occurring in the MDS. This is because the MDS region contains large beta functions, which introduce a significant natural chromaticity. To avoid affecting the bunching and demodulation of the electron beam, no additional sextupoles are added to correct for the natural chromaticity in this region. Instead, the natural chromaticity is corrected by increasing the gradient of the chromatic sextupoles in the arc.

## 3. Reversible ADM simulations

**Figure 8:**
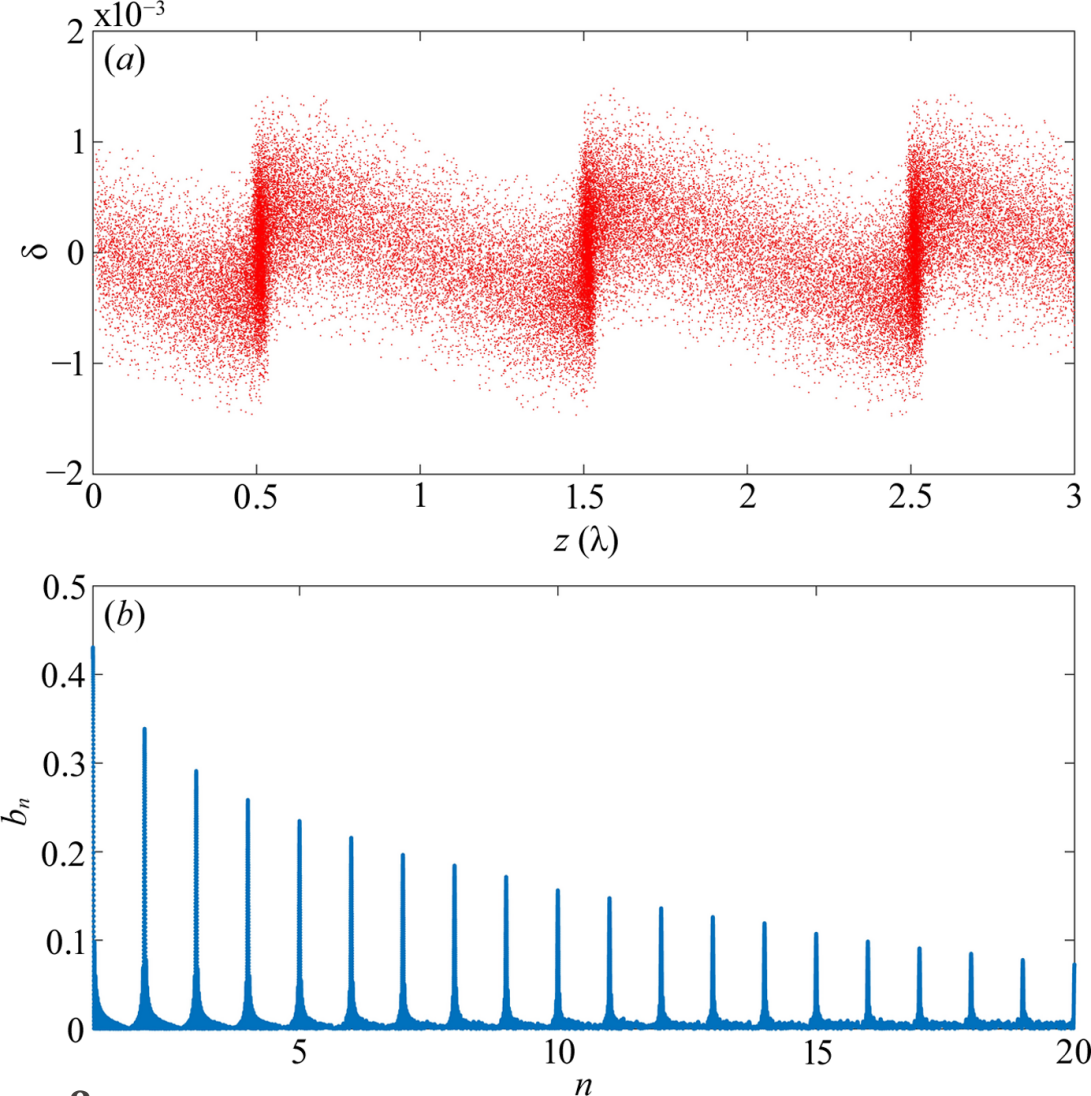
(*a*) Longitudinal phase space distribution of the electron beam at the radiator and (*b*) the corresponding bunching factor.

In the following, we demonstrate the performance of the ADM scheme through simulation using the *ELEGANT* code (Borland, 2000 ▸). The main parameters for the ADM transport line are listed in Table 2 ▸, and the emittance and energy spread parameters of the electron beam are listed in Table 1 ▸. The electron beam first passes through a vertical dipole, with a length of 0.2 m and a bending angle of 27.45 mrad, generating vertical angular dispersion. Subsequently, the electron beam passes through a modulator, modulating its energy with an amplitude approximately equal to 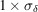. Note that the energy modulation is assumed to be an ideal sine-wave modulation. Additionally, it is assumed that the transverse size of the laser is much larger than the transverse beam size, and the modulation is homogeneous in the transverse plane. The laser pulse width is also assumed to be larger than the bunch length. Furthermore, it is assumed that the Rayleigh length of the laser is longer than that of the undulator and that the higher-order terms of the laser field are neglected. After modulation, the electron beam passes through a dogleg, which is composed of two dipoles with opposite deflection directions connected by a drift. This process converts the energy modulation into density modulation. Finally, the beam passes through a radiator, by which the high-harmonics radiation is generated. The longitudinal phase space distribution of the electron beam at the entrance of the radiator is shown in Fig. 8 ▸(*a*), and its bunching factor is shown in Fig. 8 ▸(*b*).

**Figure 9:**
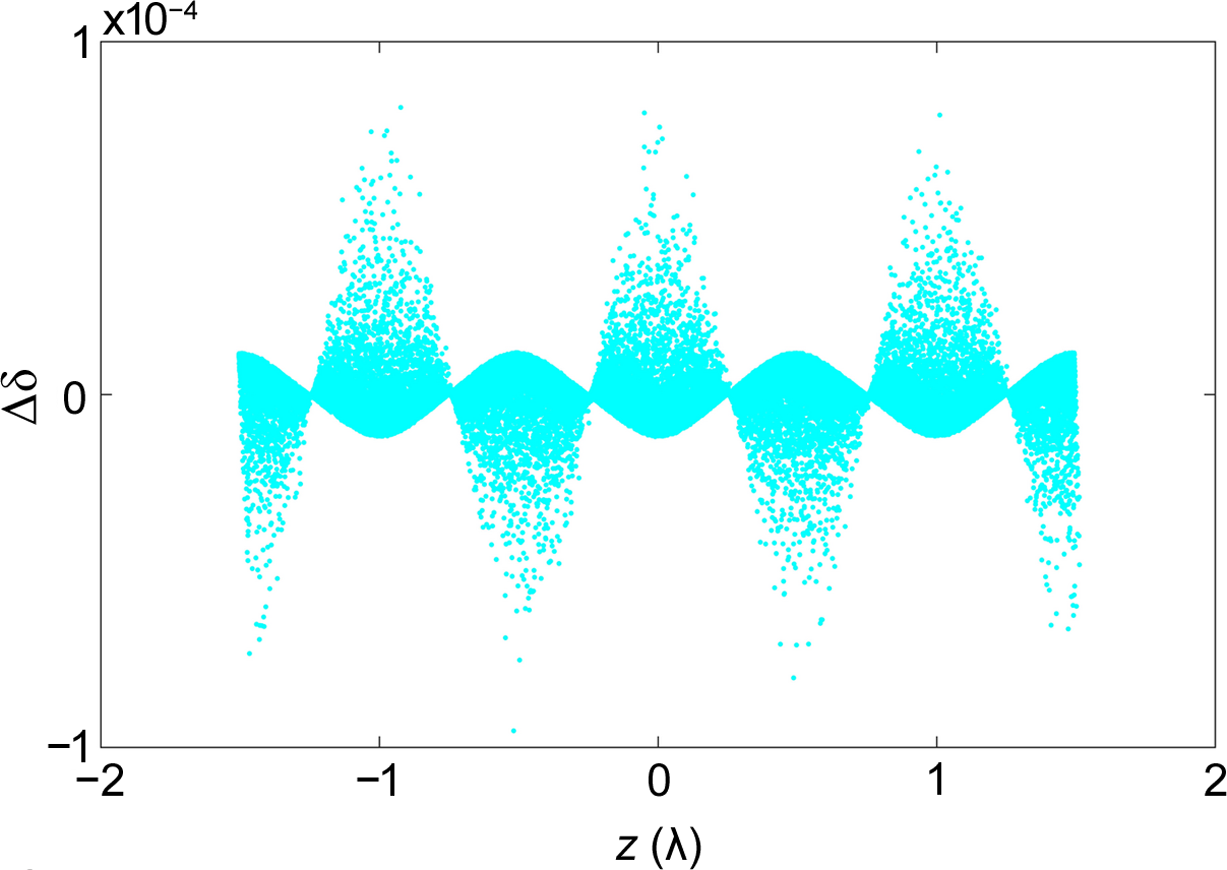
Residual energy modulation after demodulation.

**Figure 10:**
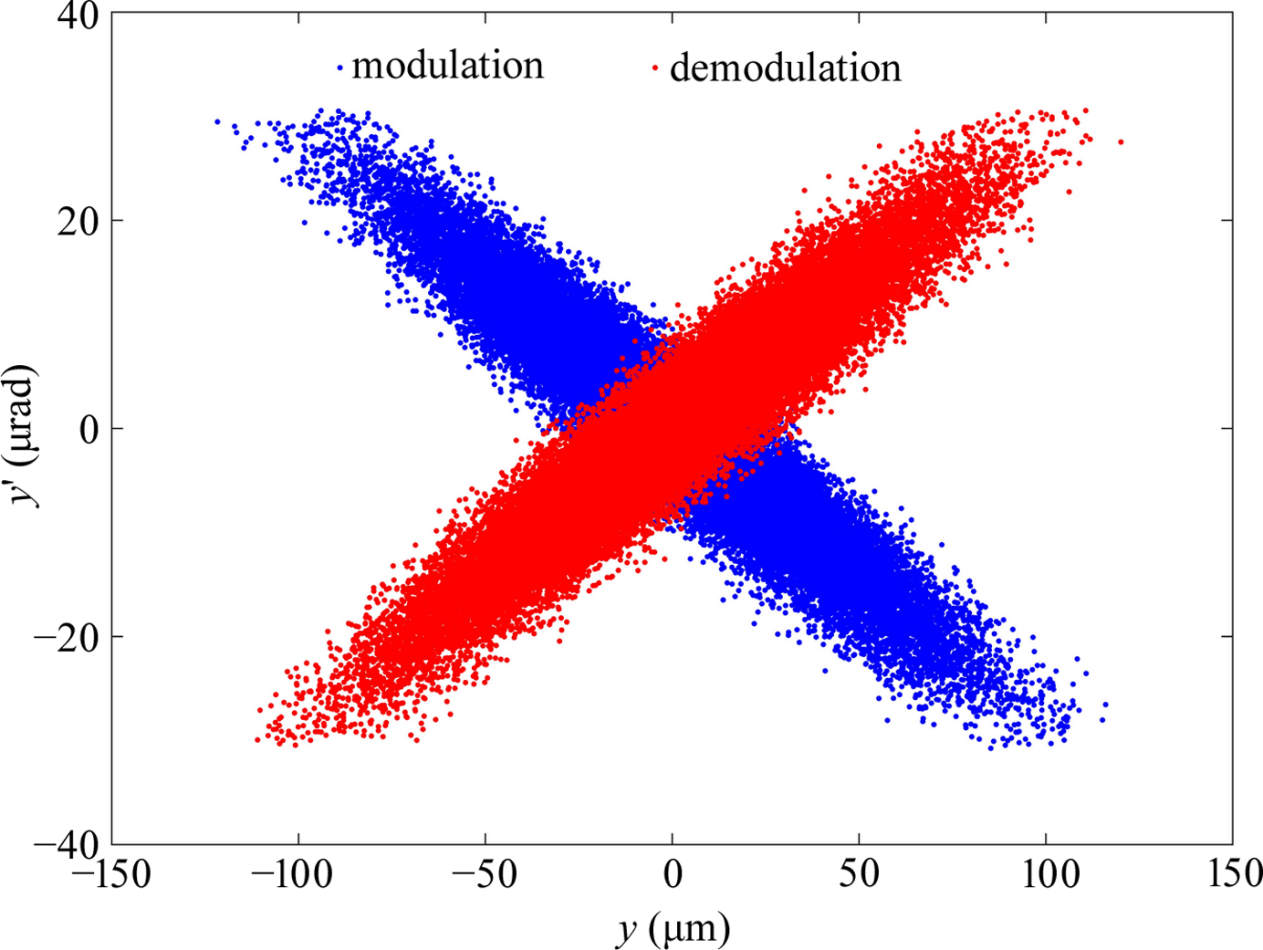
Vertical phase space distribution at the modulator and demodulator.

After being radiated by the radiator, the electron beam passes through the demodulator. The energy modulation can be canceled by a laser with a π phase different from the modulator as long as the longitudinal position shifts of the electrons relevant to its initial coordinate are zero, thereby restoring the initial state of the electron beam. However, due to the influence of various high-order transport effects, such as *T*_511_, *T*_521_, *T*_522_, *T*_566_*etc.* this cancelation is not complete. The energy spread increases intuitively. The vertical emittance also increases, as 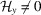 in MDS 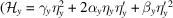, where α_*y*_, β_*y*_, η_*y*_, 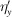 and γ_*y*_ are Twiss parameters). Since the vertical emittance is very small, the percentage of the increasement will be large. The increases in the vertical emittance and energy spread of a single pass after demodulation of the electron beam are 11% and 0.02%, respectively, for our best optimization. The horizontal direction is regarded as an irrelevant plane and is not considered here. Fig. 9 ▸ shows the residual energy modulation of the electron beam after demodulation. In contrast to Jiang *et al.* (2022 ▸), the MDS in this paper is located in the LSS of the storage ring rather than the bypass line. Therefore, adding several sextupole magnets to optimize high-order terms is not feasible as it would destroy the nonlinear dynamics of the storage ring. Fig. 10 ▸ shows the vertical phase space distribution at the modulator and demodulator. At the modulator, the Twiss parameter α_*y*_ = −5.52 × 10^−2^ and β_*y*_ = 21.10 m. Due to symmetry, β_*y*_ at the demodulator is the same as that at the modulator and α_*y*_ is the opposite to that at the modulator. Although the vertical emittance growth calculated by the statistics of the phase space particle beam distribution is 11%, it cannot be easily observed in Fig. 10 ▸.

**Figure 11:**
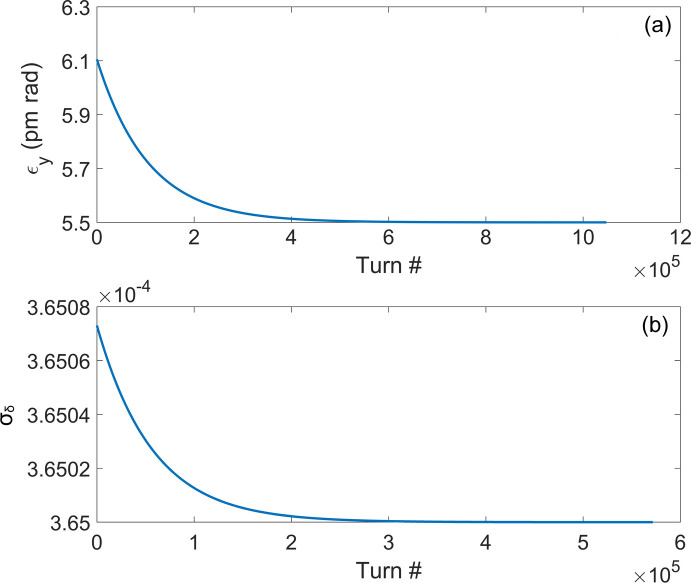
Evolution of vertical emittance and energy spread of the electron beam with the number of turns after a single-energy modulation and demodulation. The range of the horizontal axis is 5 times the vertical and longitudinal damping time.

The growth of the vertical emittance and energy spread of the electron beam after a single-energy modulation and demodulation will be damped in the storage ring according to the following equations (Di Mitri *et al.*, 2021 ▸; Emma & Raubenhemier, 2001 ▸):
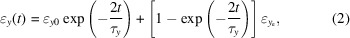

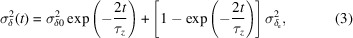
where ɛ_*y*0_ and ɛ_*y*e_ are the vertical emittances after demodulation and at the equilibrium state, respectively; σ_δ0_ and σ_δe_ are the energy spreads after demodulation and at the equilibrium state, respectively; τ_*y*_ and τ_*z*_ are the damping times in the vertical and longitudinal planes, respectively. Based on equations (2) and (3), Fig. 11 ▸ shows the evolution of the vertical emittance and energy spread of the electron beam with the number of turns after a single-energy modulation and demodulation, and it can be seen that the vertical emittance needs about 6 × 10^5^ turns to reach the equilibrium state, and the energy spread needs about 3 × 10^5^ turns to reach the equilibrium state. Therefore, the repetition rate of the coherent radiation will be determined by the damping of the vertical emittance, and it will be about 3 Hz in a single bunch. There are 280 buckets in the ring with a 500 MHz RF system, and 200 of them can be used to fill electron bunch. For the single-bunch-modulation mode, the repetition rate of the coherent radiation is 600 Hz.

## 4. Enhancing the radiation damping and increasing the repetition rate

Increasing the electron beam energy can effectively enhance damping and reduce the damping time, as the damping time is inversely proportional to the cube of electron beam energy. At the same time, the addition of a DW can also effectively enhance the radiation damping.

**Figure 12:**
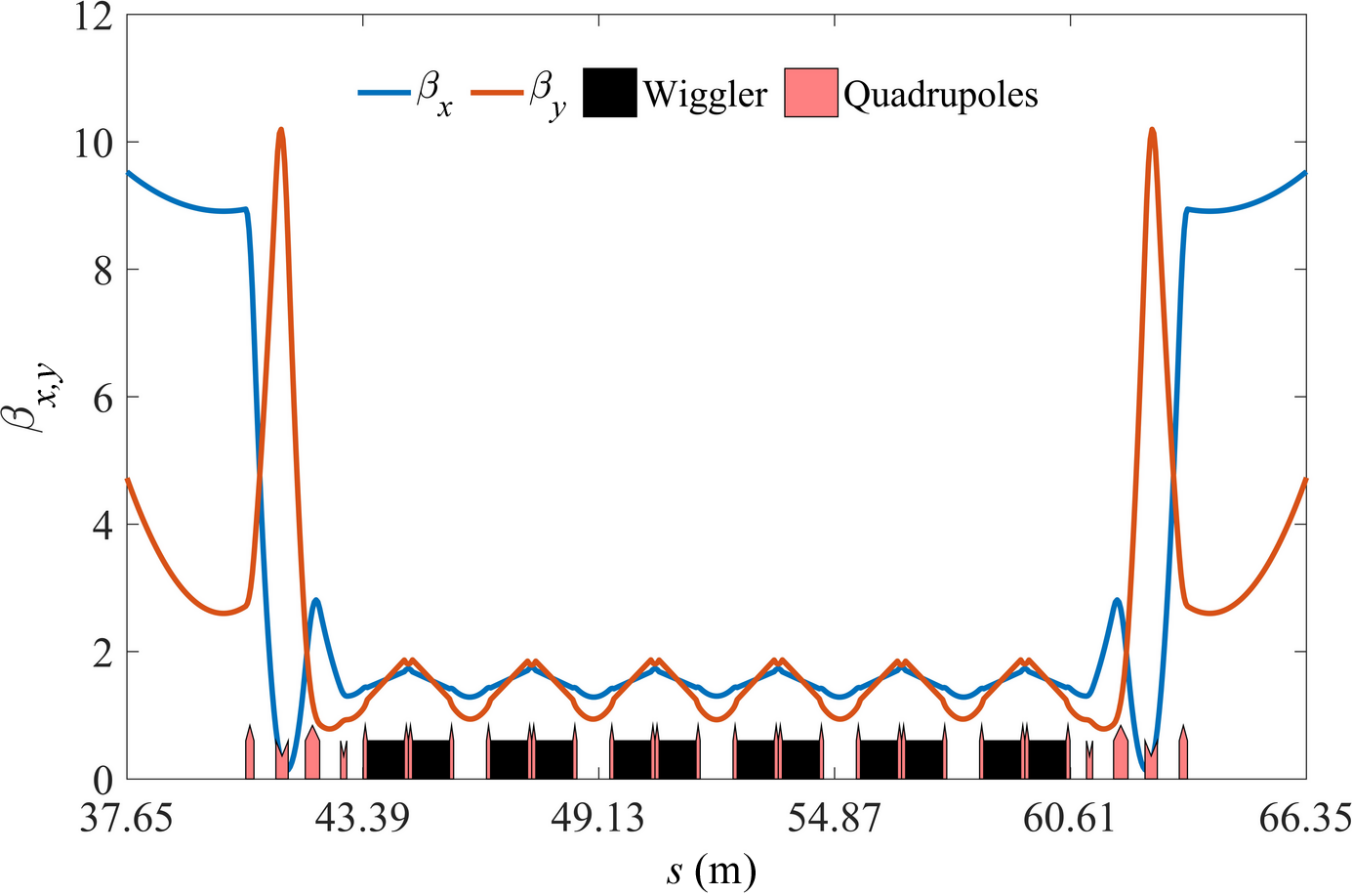
Optical functions for the DW section.

**Figure 13:**
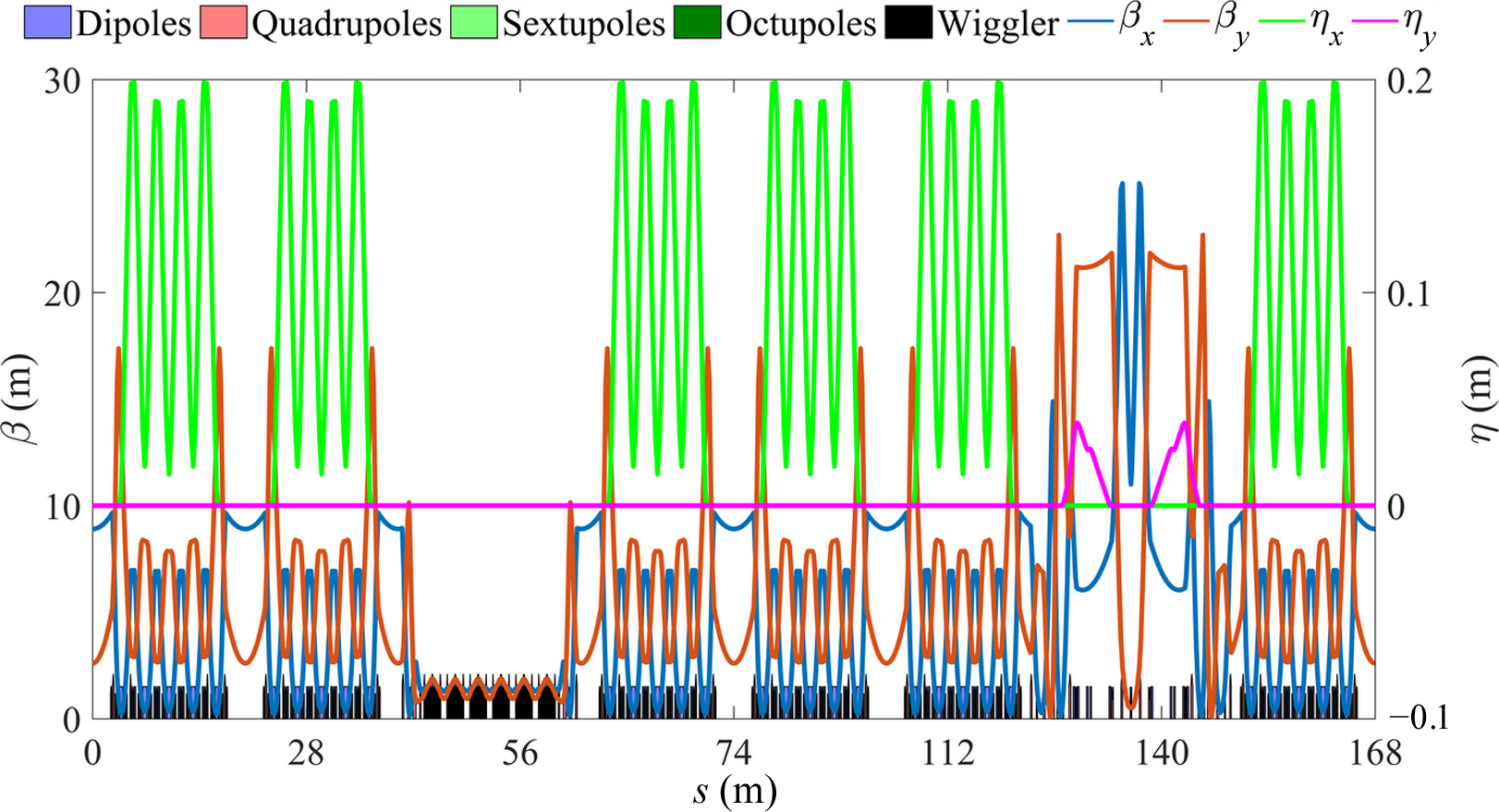
Optical functions for the full ring after adding DW.

The magnets of the ring preserve the capability to ramp to 800 MeV. Two sets of DWs with a peak magnetic field of 4.53 T and a period length of 56 mm were added. The design principle of the DW has been presented by Jiang *et al.* (2022 ▸). To prevent any negative impact on the bare ring’s nonlinear phase cancelation strategy, the DW section is designed with a phase advance of (4π, 4π) in both transverse planes. Fig. 12 ▸ shows its linear optical function and Fig. 13 ▸ displays the linear optics of the full ring after the DW section is added; Table 3 ▸ presents the full ring beam parameters.

As shown in Table 3 ▸, after increasing the electron beam energy to 800 MeV and adding the DW, the damping time of the electron beam in the vertical direction was 8.31 ms, which is a reducton of 1/14 of the original time. However, since the vertical emittance of the equilibrium electron beam decreases from 5.5 pm rad to 1.6 pm rad and the energy spread increases from 3.65 × 10^−4^ to 7.80 × 10^−4^, the relative growth rate of the vertical emittance will increase while the growth rate of the energy spread will decrease, and the growth rate of the vertical emittance and energy spread after a single modulation and demodulation is obtained from simulations will be 26% and 0.005%, respectively.

**Figure 14:**
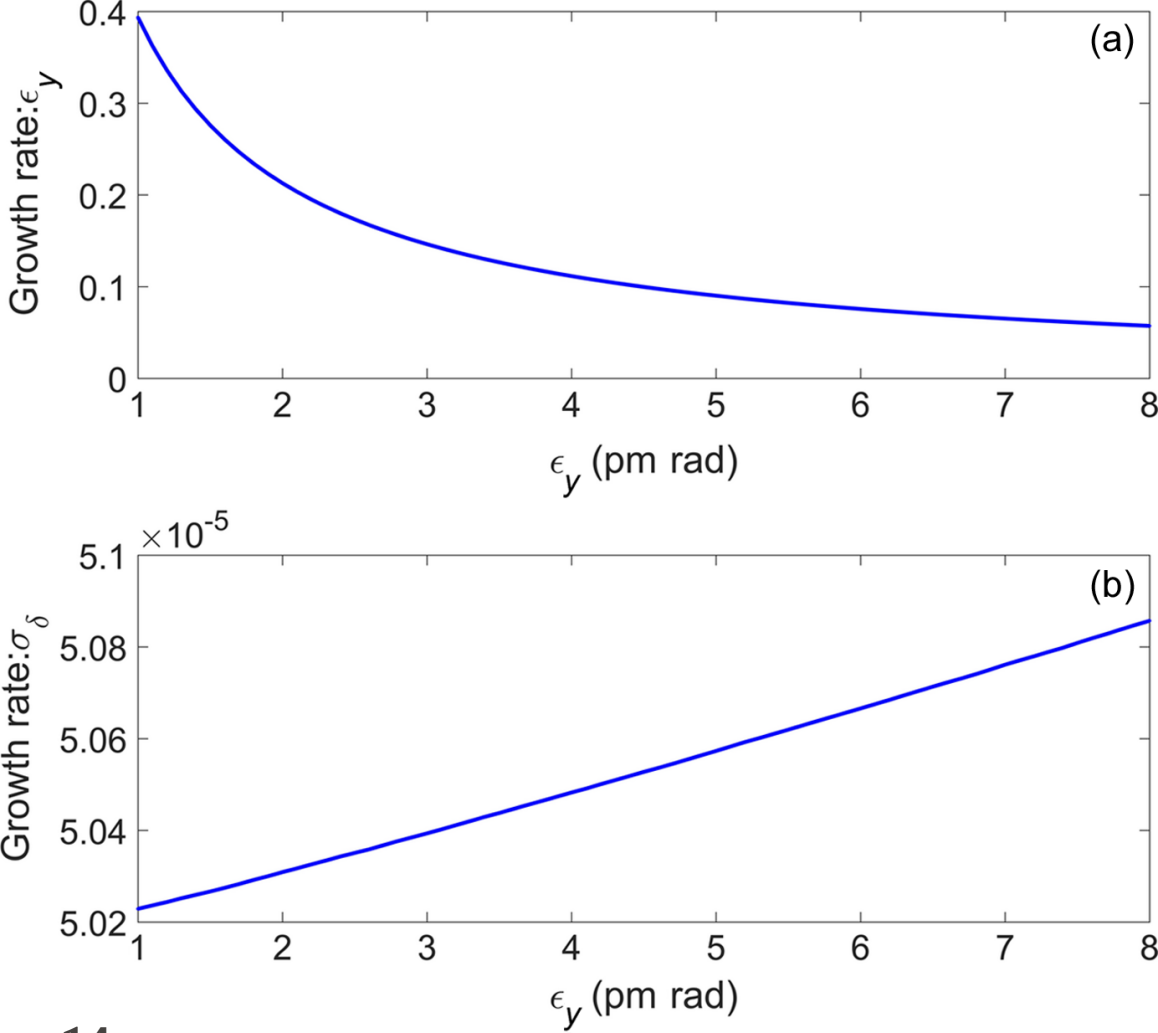
Evolution of the growth rates of (*a*) vertical emittance and (*b*) energy spread with initial vertical emittance after a single-energy modulation and demodulation.

**Figure 15:**
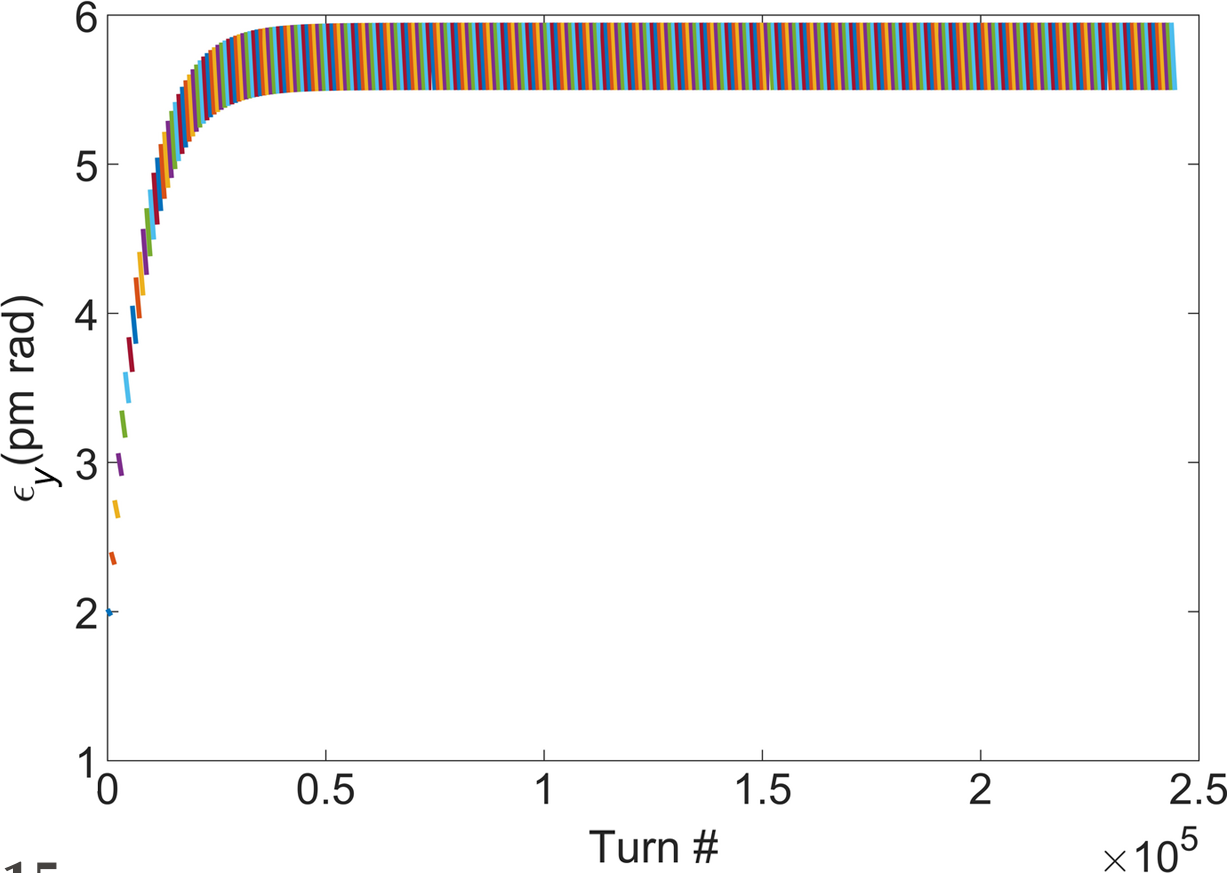
Vertical emittance evolution with the number of turns. The electron beam is damped every 815 turns before the next modulation, and the figure shows the results for only 300 modulations.

Note that the energy modulation amplitude decreases from a value of 1.02 to a value of 0.48, as the energy spread of the electron beam increases from 3.65 × 10^−4^ to 7.80 × 10^−4^. The energy spread of the electron beam is fixed at 7.80 × 10^−4^, and the growth of the vertical emittance and the energy spread of a single turn of the electron beam after one modulation and demodulation are simulated at different initial vertical emittances. The results are presented in Fig. 14 ▸, illustrating that the growth rate of the vertical emittance decreases significantly with the increase of the initial vertical emittance. Additionally, the growth rate of the energy spread is essentially negligible. Based on equations (2) and (3), as well as Fig. 14 ▸(*a*), the vertical emittance of the electron beam can be made to reach a new equilibrium state by iterating the damping process. The value of this new equilibrium state is set to be 5.5 pm rad, as the bunching factor of the electron beam is still good at this vertical emittance, as shown in Fig. 8 ▸(*b*). As demonstrated in Fig. 15 ▸, the electron beam is damped every 815 turns before the next modulation. The figure shows only 300 electron beam modulations, which allows for the observation that the vertical emittance of the electron beam has reached a new equilibrium state. In this state, the vertical emittance is 5.5 pm rad and its growth rate is 8.23%. Fig. 15 ▸ illustrates that the electron beam achieves a new equilibrium state after every 815 turns of modulation. This implies that the repetition rate of a single bunch is 2.19 kHz. There are 280 buckets in the ring with a 500 MHz RF system and 200 of them can be used to fill the electron bunch. For single-bunch-modulation mode, the repetition rate of the coherent radiation is 438 kHz.

**Figure 16:**
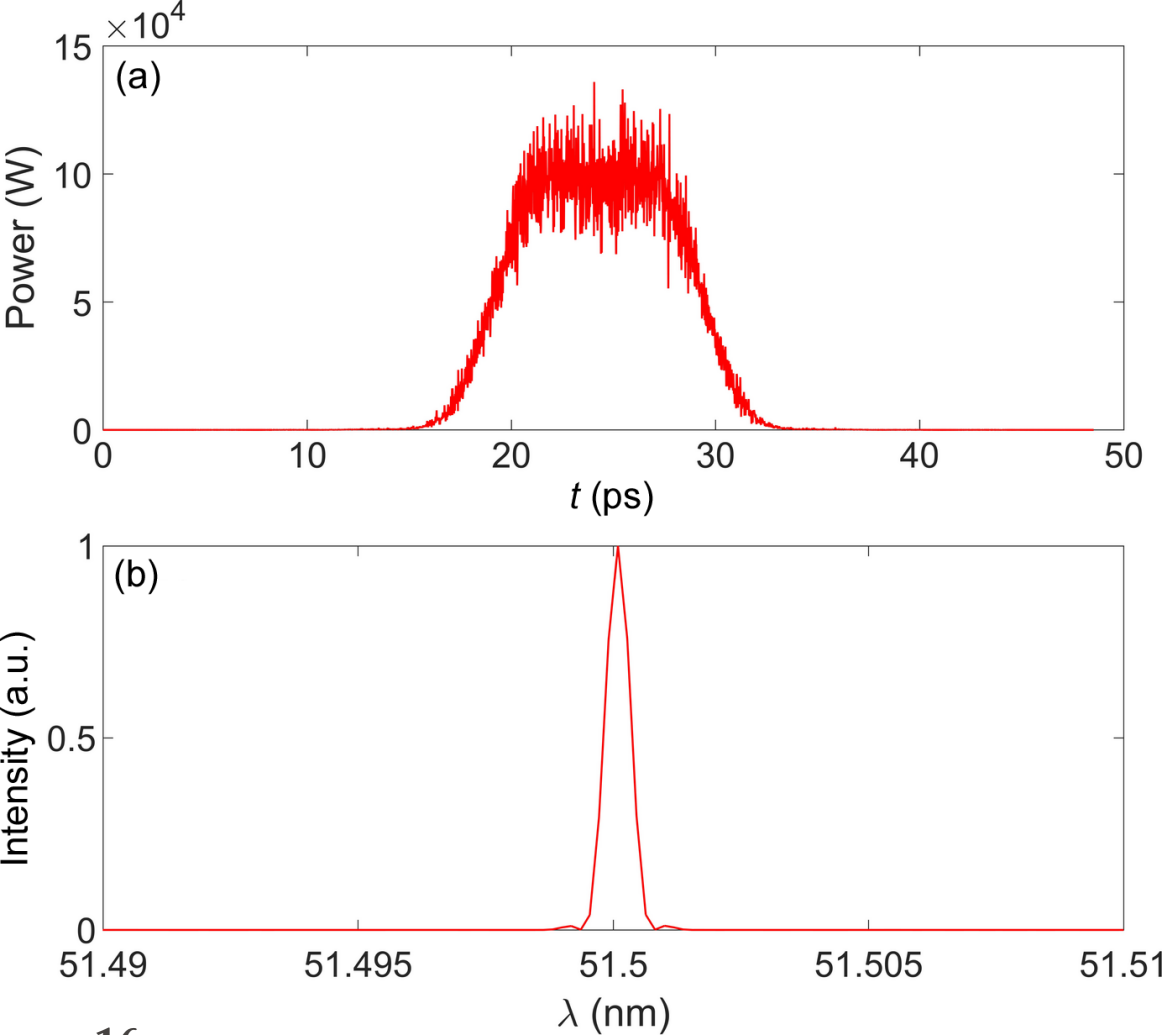
Performance of the radiation pulse emitted by the radiator: (*a*) output power and (*b*) single-shot spectrum.

The longitudinal profile and the corresponding spectrum of a single radiation pulse simulated by the *GENESIS* code (Reiche, 1999 ▸) are shown in Fig. 16 ▸. The simulation was performed with a beam current of 200 mA, and 200 buckets were selected to be uniformly filled, resulting in single bunch charge of 0.56 nC. The wavelength of the modulated laser is 1030 nm, with a peak power of 27 MW and a pulse energy of 540 µJ. The radiator is a 2 m-long undulator with a period length of 60 mm, which produces a single pulse energy of about 1.05 µJ. With a repetition rate of 438 kHz, the average power is calculated to be approximately 0.46 W. Two radiators are installed in MDS with a canted angle of 21.5 mrad in the vertical plane, thereby enabling the proposed storage ring to attain a total-output average power of 0.92 W. In addition, the spectral bandwidth in terms of the full width at half-maximum is only 0.27 meV, which is quite close to the Fourier transform limit.

## 5. Conclusions

This paper integrates the ADM modulation and demodulation scheme into the LSS of the storage ring for the first time and provides a detailed lattice design of the test facility. At the same time, it simulates the performance of ADM and the effect of demodulation. By increasing the energy of the electron beam to 800 MeV and adding a DW, we can achieve 438 kHz coherent radiation. Although the repetition rate of coherent radiation is lower than that in Jiang *et al.* (2022 ▸), this scheme reduces the challenges associated with this hardware system, especially for the bypass injection and extraction system.

The intra-beam scattering effects in the storage ring and the nonlinear optimization of the full ring after adding a DW are not discussed here, but will be the next step of our work.

This work provides a possible research direction for the development of the post-diffraction-limited storage ring (DLSR), that is, combining the transverse coherence of the DLSR with the longitudinal coherence of ADM to produce fully coherent radiation in a storage ring. Moreover, the repetition rate of the storage ring coherent light source can be further improved by adding demodulation and a DW.

## Figures and Tables

**Table 1:** Storage ring parameters

Beam energy	600 MeV
Circumference	168 m
Lattice type	6 × 5BA
Tunes *x*/*y*	16.246/6.178
Geometric horizontal emittance	498 pm rad
Geometric vertical emittance	5.5 pm rad
Relative energy spread	3.65 × 10^−4^
Momentum compaction factor	3.67 × 10^−4^
Energy loss per turn	5.73 keV
Damping times *x*/*y*/*z*	100.58/117.37/64.03 ms
RF frequency	499.6539 MHz
RF voltage	0.25 MV
Harmonic number	280
R.m.s. bunch length	1.37 mm

**Table 2:** Main parameters employed in the simulations

Laser wavelength	1030 nm
Bending angle of *B*_0_	27.45 mrad
Length of dipoles	0.2 m
*R*_56_ of dogleg	−4.38 × 10^−4^ m
Dispersion of dogleg	15.2 mm
Distance between two dipoles in the dogleg	0.25 m
Amplitude of energy modulation	1.02

**Table 3:** Storage ring parameters for 800 MeV

	Without DW	With DW
Beam energy (MeV)	800	800
Beam current (mA)	200	200
Circumference (m)	168	168
Lattice type	6 × 5BA	6 × 5BA
Tunes *x*/*y*	16.246/6.178	18.246/8.178
Geometric horizontal emittance (pm rad)	885	242
Geometric vertical emittance (pm rad)	9.57	1.60
Relative energy spread	4.87 × 10^−4^	7.80 × 10^−4^
Momentum compaction factor	3.67 × 10^−4^	3.60 × 10^−4^
Energy loss per turn (keV)	18.10	107.88
Damping times *x*/*y*/*z* (ms)	42.45/49.53/27.02	8.09/8.31/4.22
